# Feasibility and Safety of Transbronchial Lung Cryobiopsy for Diagnosis of Acute Respiratory Failure with Mechanical Ventilation in Intensive Care Unit

**DOI:** 10.3390/diagnostics12122917

**Published:** 2022-11-23

**Authors:** Chih-Hao Chang, Jia-Shiuan Ju, Shih-Hong Li, Shao-Chung Wang, Chih-Wei Wang, Chung-Shu Lee, Fu-Tsai Chung, Han-Chung Hu, Shu-Min Lin, Chung-Chi Huang

**Affiliations:** 1 Department of Thoracic Medicine, Chang Gung Memorial Hospital at Linkou, Taoyuan 333, Taiwan; 2 College of Medicine, Chang Gung University, Taoyuan 333, Taiwan; 3 Division of Pulmonary and Critical Care Medicine, Department of Internal Medicine, New Taipei Municipal Tucheng Hospital, New Taipei 236, Taiwan; 4 Department of Medical Imaging and Intervention, New Taipei Municipal Tucheng Hospital, New Taipei 236, Taiwan; 5 Department of Medical Imaging and Intervention, Chang Gung Memorial Hospital at Linkou, Chang Gung University, Taoyuan 333, Taiwan; 6 Department of Pathology, Chang Gung Memorial Hospital at Linkou, Taoyuan 333, Taiwan

**Keywords:** ARDS, bronchoscopy, critical care, TBLC, lung

## Abstract

Background: Acute hypoxemic respiratory failure is common in intensive care units (ICUs). Lung biopsies may be required to make a definitive diagnosis in patients with unknown etiologies. The feasibility of transbronchial lung cryobiopsy is undetermined in patients with respiratory failure in the ICU. Methods: Patients who underwent bronchoscopy examinations with transbronchial lung cryobiopsy (TBLC) between July 2018 and December 2019 were retrospectively analyzed through medical chart review. The procedures were performed by well-experienced interventional pulmonologists. Results: Ten patients underwent bronchoscopy examinations with TBLC in the ICU at Chang Gung Memorial Hospital during the study period. In all patients, the diagnosis was made via pathological analysis. One patient developed pneumothorax and required chest tube placement after the procedure. None of the patients had bleeding requiring blood transfusion, and no deaths were directly related to the procedure. Conclusions: TBLC is a feasible technique to obtain lung pathology in patients with acute respiratory diseases of unknown etiologies. While the complication rate may be acceptable, the procedure should be performed by experienced interventional pulmonologists. However, airway blockers and fluoroscopy are highly recommended when used according to the current guideline. We do not encourage TBLC to be performed without having airway blockers available at the bedside.

## 1. Introduction

Acute hypoxemic respiratory failure is a common and critical condition in intensive care units (ICUs) [1,2]. The causes of acute hypoxemic respiratory failure vary, and diagnosis is usually based on microbiological and radiologic findings. Sputum collection, tracheal aspiration, and bronchoalveolar lavage are used to obtain a definitive diagnosis of acute respiratory failure (ARF) with bilateral lung infiltration [3]. When histological diagnosis of acute respiratory failure is needed, transbronchial lung biopsy to obtain histological specimens is the primary endoscopic technique adopted in this particular clinical setting, but it must be evaluated on a case-by-case basis, following guidelines [4,5,6]. In cases with unknown etiology, surgical lung biopsies provide further aid in determination of diagnosis and may provide treatment alternatives [7,8,9]. A meta-analysis of 22 studies showed that patients with acute respiratory distress syndrome (ARDS) experienced a change in management after surgical lung biopsy [10]. However, the complication rate of surgical lung biopsy is up to 24% in patients with ARDS.

Cryobiopsy is a new technique used for the diagnosis of pulmonary diseases. Bronchoscopists could get larger biopsy specimens by using cryoprobe rather than traditional forceps biopsy [11,12]. The current applications of bronchoscopic cryobiopsy are biopsy of endobronchial tumors, diffuse interstitial lung diseases, and peripheral lung nodules [13]. Some authors suggest that transbronchial cryobiopsy is a potential alternative to surgical lung biopsy in diffuse parenchymal lung disease diagnosis [14]. Recent guidelines suggest surgical lung biopsy should even be replaced with TBLC in eligible patients [15].

Gerard and his colleague enrolled 51 patients with non-resolving acute respiratory distress syndrome and concluded that open lung biopsies could identify a steroid-sensitive pathology [16]. With recent advances in bronchoscopy, transbronchial lung cryobiopsy is used to diagnose diffuse lung disease [17,18,19]. However, the use of TBLC in ARF with mechanical ventilation has seldom been reported. Compared to surgical lung biopsy, TBLC is a less invasive method to obtain a specimen. A recent literature review of 25 patients with respiratory failure who underwent TBLC concluded that TBLC is an acceptable procedure [20]. Intensive care physicians and pulmonologists are concerned about the safety and diagnostic efficacy of lung biopsies performed in the ICU. Pneumothorax and bleeding are the most common complication in transbronchial lung biopsy [21] and persistent air leaks are most common in surgical lung biopsy [22]. Herein, we report the feasibility and safety of TBLC in ARF patients with mechanical ventilation in the ICU. 

## 2. Methods

### 2.1. Study Population

We performed this retrospective study at Linkou Chang Gung Memorial Hospital, which is a tertiary referral medical center in Taiwan. Data were collected for all patients > 20 years of age who underwent bronchoscopy procedures with cryotechnology between July 2018 and December 2019. Information was collected through medical chart review. Only patients with acute respiratory failure and mechanical ventilation who underwent bronchoscopic cryobiopsies were included. All patients were also intubated, and there were no patients with noninvasive ventilation. Patients who underwent bronchoscopy examinations with cryotechnology in the procedure room were excluded.

Bronchoscopic cryobiopsy was considered when the cause of hypoxemic respiratory failure was unknown and noninfectious etiology was possible. Medical records were reviewed and the following detailed clinical data were recorded: age, sex, underlying diseases, laboratory data, PaO_2_/FiO_2_ ratio, positive end-expiratory pressure (PEEP), tidal volume, pathological diagnosis, procedure-related complications (i.e., pneumothorax or major bleeding), and outcomes. This study was approved, and the need for informed consent was waived, by the Institutional Review Board of the Chang-Gung Medical Foundation (CGMH IRB No. 202102026B0).

### 2.2. Bronchoscopy

Bronchoscopy was performed using a flexible bronchoscope (BF-P240 or BF-40; Olympus, Tokyo, Japan) through the endotracheal tube. All the patients were deeply sedated by midazolam with or without cisatracurium. To prevent temporary hypoxemia during the fibreoptic bronchoscopy procedure, the FiO_2_ (fraction of inspired oxygen) of the mechanical ventilation was adjusted to 1.0 before the procedure. 

Heart rate, blood pressure, respiratory rate, and oxygen saturation were continuously monitored during the bronchoscopy procedure in the ICU. Bronchoscopy was performed at the bedside and without fluoroscopic guidance. All patients underwent chest computed tomography (CT) before the procedure. The location for tissue sampling was determined based on the chest CT findings reviewed by bronchoscopists and the intensivist. The biopsy lobe chosen was entirely white-out in CT scans. Bronchoscopists were experienced, and the results of TBLC in lung parenchymal lesions have been reported in a previous study [23].

Cryobiopsy was performed using a 1.9 mm flexible cryoprobe (Erbokryo CA, Erbe, Germany) with carbon dioxide as the cryogen with a temperature of −70 °C at the probe tip. Because fluoroscopy guidance is not available in the ICU, the cryoprobe was withdrawn 1–1.5 cm when the cryoprobe met a resistant point. Probe cooling time was 4–6 s. After cooling, the cryoprobe was immediately retracted using the bronchoscope. Frozen biopsy specimens were thawed in normal saline and fixed in formalin. Finally, the bronchoscope was reintroduced to confirm airway status [23,24]. Bronchial blockers (e.g., Fogarty balloon) were not available during the study period. Before bronchoscopy, prothrombin time, activated partial thromboplastin time, and platelet count were checked to exclude the possibility of bleeding tendency. After biopsy, a bronchoscope was inserted to wedge into the possible bleeding site. The status of possible bleeding was carefully checked by direct observation via bronchoscope to ensure there was no active bleeding from the biopsy site. Two to four samples were planned to be collected from each patient.

### 2.3. Statistical Analyses

Continuous variables were expressed as mean ± standard deviation and categorical variables as frequency and percentage. All statistical analyses were performed using MedCalc, Version 12.5 (MedCalc Software, Ostend, Belgium). 

## 3. Results

We retrospectively identified 10 patients who underwent bronchoscopy examinations with TBLC in the ICU at Chang Gung Memorial Hospital between July 2018 and December 2019. The baseline characteristics of the 10 patients are presented in Table 1. All patient ratios of the partial pressure of oxygen in arterial blood (PaO_2_) to the inspired oxygen fraction (FiO_2_) were less than 300 mmHg. The mean PaO_2_/FiO_2_ ratio was 209.78 ± 60.77 mmHg, and the mean FiO_2_ was 43.88 ± 8.94. All 10 patients underwent chest CT scans and bronchoalveolar lavage (BAL) before the cryobiopsy procedure. There was no definite pathogen found via BAL in any of the 10 patients. The BAL and cryobiopsy were not done in the same bronchoscopy procedure. Because BAL was done in advance, the days from admission to biopsy were 7.5 ± 4.95. Five patients (50%) died during hospitalization in the ICU because of persistent hypoxemia and multi-organ failure. The deaths occurred at a mean of 18.2 ± 10.1 days after cryobiopsy. The other five patients (50%) were alive and discharged from the hospital at a mean of 22.4 ± 8.1 days after cryobiopsy. Two patients had a history of cancer; one had been diagnosed with lung adenocarcinoma, and the other had diffuse large B-cell lymphoma post-bone marrow transplantation. 

**Figure 1:**
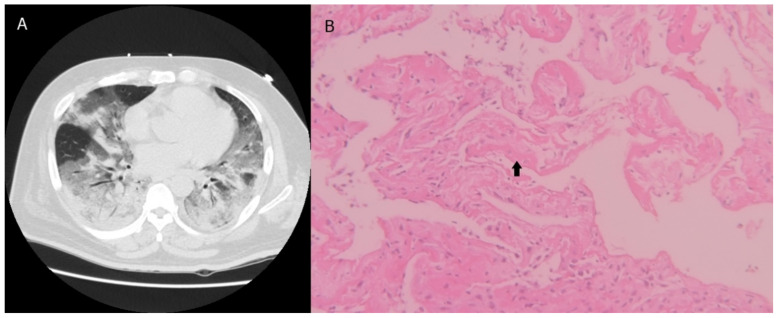
(**A**) The transverse unenhanced chest CT image showed dependent and peribronchovascular consolidations as well as diffuse heterogeneous ground glass opacities in bilateral lungs. (**B**) The pathology result showed diffuse alveolar damage patterns. The alveolar walls were thickened by organizing loose connective tissue. Hyaline membranes were seen (arrow) (hematoxylin and eosin stain, 200×).

**Figure 2:**
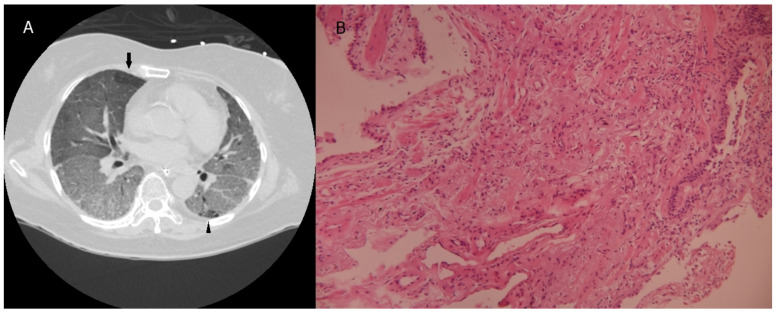
(**A**) The transverse unenhanced chest CT image showed diffuse ground glass opacities and reticulations in bilateral lungs. There were foci of air trapping in the peripheral right upper lung (arrow) and subpleural honeycombing in the left lower lung (arrowhead). (**B**) The lung parenchyma showed fibrosis (hematoxylin and eosin stain, 100×).

Table 2 shows the biopsy location and pathological diagnoses for the 10 patients. The average number of biopsies was 2.80 ± 1.40. In the histological biopsies, the mean specimen diameter was 0.56  ±  0.21 cm, and the mean biopsy area was 0.17  ±  0.08 cm squared. All the biopsies had pathological diagnoses. Four patients had diffuse alveolar damage (Figure 1), which is compatible with the typical pathology seen in acute respiratory distress syndrome. Two patients without a cancer history were diagnosed with lung cancer after TBLC; one had lung adenocarcinoma, and the other had lung squamous cell carcinoma. Three patients had lung fibrosis; one was diagnosed with connective tissue disease-related interstitial lung disease (Figure 2), and another was diagnosed with chronic inflammation. Biopsy changed the management in seven of the 10 patients after TBLC. Two patients were reassessment for lung cancer treatment. Intravenous corticosteroids were started in four patients. Graft-versus-host disease was ruled out after biopsy in one patient with previous bone marrow transplantation. A patient diagnosed with systemic lupus erythematosus-related interstitial lung disease received antifibrotic treatment. Besides treatment alteration, graft-versus-host disease was ruled out after biopsy in one patient with previous bone marrow transplantation.

Procedure-related complications were recorded in the charts under review, including procedural reports, radiologic reports, and medical records (Table 3). No patients had severe bleeding that required local epinephrine, blood transfusion, angiography, or surgery. After the procedure, one patient developed pneumothorax and required chest tube insertion: they also had significant hypoxemia (oxygen saturation <90% on FiO_2_ 1.0) that improved after chest tube insertion. No significant changes were found in FiO_2_ or ventilator parameters in the other nine patients. None of the patients had ventricular arrhythmia, hypotension, hemodynamic instability required vasopressors, or biopsy-related death (death occurring within 24 h of the procedure). 

## 4. Discussion

Our study included all patients from current and previous studies, making it one of the largest case series to assess the feasibility and safety of TBLC in ARF and ARDS. In ICUs, acute hypoxemic respiratory failure is common and etiologies are heterogeneous [25]. Bronchioalveolar lavage is an invasive but useful tool for intensivists to differentiate the cause of hypoxemic respiratory failure [26]. However, despite advances in diagnostic testing, 10% of patients have no identified ARDS risk factors before disease onset [27]. Another study reviewed 665 patients with ARDS who received invasive mechanical ventilation in the ICU, and the prevalence of ARDS without common risk factors was 7.5% [28].

Some researchers have attempted to identify the cause of ARF using surgical lung biopsy [29]. Biopsy or autopsy to obtain a specimen can provide additional information about the disease, especially without a definitive diagnosis. Gerard et al. attempted to identify a steroid-sensitive pathology in patients with non-resolving ARDS using lung biopsy [16]. During the COVID-19 pandemic period, post-mortem biopsy has helped identify the diagnosis, pathology, and etiology of respiratory failure [30,31]. 

Fluoroscopy is useful for identifying peripheral lung lesions and ensures that the bronchoscope does not contact the visceral pleura. Thus, fluoroscopy could prevent the development of iatrogenic pneumothorax during bronchoscopy procedures. The current guidelines also recommend fluoroscopy to reduce complications; however, it is not always available in the ICU. For TBLC in diffuse parenchymal lung disease, confirming lesion locations through X-ray imaging and radial endobronchial ultrasonography is another option [32]. In hospitals without fluoroscopy, radial endobronchial ultrasonography-guided TBLC may be beneficial [23]. To prevent pneumothorax development, some bronchoscopists suggest that the cryoprobe should be withdrawn approximately 1 cm during cryobiopsy for diffuse lung disease. In our hospital, radial probe endobronchial ultrasound was available in the procedure room to localize peripheral lung lesions. Whether radial endobronchial ultrasonography combined with transbronchial lung cryobiopsy is feasible in the ICU needs further research. 

A retrospective comparative study enrolled patients with acute hypoxemic respiratory failure with unknown etiologies who underwent traditional transbronchial lung forceps biopsy or transbronchial lung biopsy [33]. The authors found that TBLC had a higher pathological diagnostic yield rate than TBLB (72.0% vs. 37.8%). Furthermore, treatment adjustment rates in patients who underwent TBLC were also higher than in those who underwent TBLB. Matta et al. performed TBLC for diffuse parenchymal lung disease at the bedside of patients with acute hypoxemic respiratory failure without fluoroscopic guidance at two tertiary hospitals [34]. After pathological diagnosis made by TBLC, 88% of patients had treatment adjustments. Bronchoscopy has been proven to have a high diagnostic yield similar to that of SLB, with a more acceptable safety profile. In the study, TBLC was also contributive and the pathological finding resulted in treatment changes.

In this study, one patient developed pneumothorax after TBLC and died 26 days after the procedure. However, the remaining patients had no bleeding events or any other major complications. Matta reported 17 transbronchial cryobiopsies in critically ill patients with acute hypoxemic respiratory failure; six patients had pneumothorax after the procedure, but no deaths were directly related to TBLC. Heras et al. reported 10 cases of TBLC for diagnosis of acute respiratory failure in the ICU [35]. The author describes no pneumothorax or biopsy-related deaths after the procedure. However, complication rates vary among hospitals. Bronchoscopists should perform TBLC meticulously to limit complications as much as possible. 

Torrego et al. reported on a multicenter, prospective, observational study conducted in three different centers [36]. All the bronchoscopists in the hospitals were well-experienced in performing TBLC. The authors revealed that TBLC is feasible in mechanically ventilated ICU patients, and these patients had no pneumothorax or death occurring within 24 h of the procedure. It is argued here, that researchers should be encouraged to find the diagnostic value of transbronchial lung cryobiopsies in patients with mechanical ventilation after the safety and feasibility of TBLC are recognized.

One of our cases in the study was diagnosed with connective tissue disease-related interstitial lung disease before acute respiratory failure. The patient had progressive pulmonary fibrosis and the rheumatologist treated them with steroids, mycophenolate mofetil, and rituximab for her connective tissue disease-related interstitial lung disease (CTD-ILD). However, the patient had a poor response to the previous medication and was admitted to the intensive care unit due to acute respiratory failure with mechanical ventilation. For patients with connective tissue disease with relatively immunocompromised status, infection, drug-related pulmonary fibrosis, or CTD-ILD cannot be confirmed after BAL. The biopsy result showed lung parenchyma severe fibrosis, and systemic lupus erythematosus-related interstitial lung disease was confirmed after multidisciplinary discussion. CTD-ILD has various features of interstitial lung abnormalities on HRCT, and it has been reported that 23% of patients with systemic sclerosis have disease progression of ILD [37]. Whether biopsy is required in CTD-ILD remains controversial. Some authors suggest that surgical lung biopsy is seldom used and rarely provides additional information on CTD-ILD [38]. Even in cases of disease progression, surgical lung biopsy is usually not recommended. TBLC may be another option for those patients with progressive pulmonary fibrosis with poor treatment response. 

In this study, two patients had malignancies before acute respiratory failure; one had a history of lung adenocarcinoma, and the other had diffuse large B-cell lymphoma. Patients with hematological malignancy or solid cancer are often relatively immunocompromised and may have an occult infection, especially when they receive anti-cancer therapy. When patients with malignancy have abnormal lung infiltration, cancer recurrence, infectious disease, drug-induced pneumonitis, radiation pneumonitis, transfusion-related acute lung injury, and graft versus host disease should be included in differential diagnoses. It is difficult for clinical physicians to diagnose the etiology of lung infiltration early and offer adequate therapy rapidly in patients with hematologic malignancies. When lung disease progresses, open lung biopsy, TBLB, and TBLC may be helpful to make a definite diagnosis [39]. In critically ill patients with cancer diagnosed with ARDS, biopsy findings have resulted in a change in therapy in 69% of patients [40]. Combined BAL and TBLB are better than BAL alone in the diagnosis of pulmonary neoplastic infiltration in patients with hematologic malignancies [41]. 

Safety is the most important issue for TBLC in the ICU. But previous literature is limited and we do not encourage TBLC in the ICU if the bronchoscopists cannot perform the procedure with adequate equipment and protocol. Heras et al. [35] treated 10 TBLC in-patients with acute hypoxemic respiratory failure of undetermined origin at the bedside in the ICU. Two bronchoscope methods were used to avoid bleeding. Another bronchoscope was used to wedge into the biopsy site to stop bleeding. Zhou et al. [20] treated four TBLC in-patients with non-resolving acute respiratory distress syndrome at the bedside in the ICU. The authors used an airway blocker to stop the bleeding. Because experience with TBLC in the ICU is limited, we suggested that interventional pulmonology specialists read the CHEST Guideline carefully [42]. Furthermore, fluoroscopy should also be used and is widely available. To perform this procedure in critically ill patients, bronchoscopists should take every precaution and use airway blockers to prevent bleeding and fluoroscopy to prevent pneumothorax.

This study had several limitations. First, the group of patients is too small to have clinical and statistical significance. This was a retrospective study, and patients who underwent TBLB were highly selected. Second, the procedure required a well-trained bronchoscopist, and procedure protocols were not similar between studies, which makes our findings difficult to generalize. It has been suggested that the learning curve for transbronchial lung cryobiopsy in diffuse lung disease is approximately 70 procedures [43]. Third, the CHEST panel report recommends the use of fluoroscopy guidance and bronchial blockers in TBLC procedures [42]. However, fluoroscopy and bronchial blockers were not available in this study. Further studies should be performed based on standard TBLC guidelines.

In conclusion, this study showed that TBLC is feasible in patients with acute respiratory failure. TBLC may have a high diagnostic yield and can lead to a reevaluation of the diagnosis as well as changes in therapeutic management. However, complications may be inevitable in TBLC, even with well-experienced bronchoscopists. However, the use of airway blockers and fluoroscopy are highly recommended, subject to the use of the current guideline. We do not encourage TBLC to be performed without having airway blockers available at the bedside.

## Figures and Tables

**Table 1:** Baseline characteristics of patients.

Characteristics	Positive Pressure at ICU (*N* = 10)
Age, years	58.2 ± 13.45
Female sex, *N* (%)	5 (50%)
Body mass index, (kg/m^2^)	26.7 ± 5.28
Active smoker, *N* (%)	2 (20%)
COPD	0
asthma	0
Interstitial lung disease	0
hypertension	1
Diabetes	1
Cancer	2
FiO_2_	43.88 ± 8.94
PaO_2_	91.68 ± 35.73
PaO_2_/FiO_2_ ratio	209.78 ± 60.77
PEEP	11.33 ± 2.83
Tidal volume, mL	403.22 ± 93.33
White blood cell count	17844.44 ± 13861.58
Hemoglobin	11.37 ± 3.11
Platelet count	198.33 ± 141.81
INR	1.26 ± 0.18
Empiric antibiotics treatment before biopsy, *N* (%)	10 (100)
Days from admission to biopsy	7.5 ± 4.95
ICU length of stay, days	19.8 ± 9.8
Hospital days	32.9 ± 18.69
Hospital mortality, *N* (%)	5 (50)

**Table 2:** Histopathological diagnoses of patients receiving cryobiopsy.

Biopsy Size	
Specimen diameter, cm	0.56 ± 0.21
Specimen area, cm^2^	0.17 ± 0.08
**Biopsy locations**	
Right upper lobe	1
Right lower lobe	1
Left upper lobe	1
Lingula	5
Left lower lobe	2
**Pathological diagnosis**	
Diffuse alveolar damage	4
Cancer	2
Fibrosis	3
Chronic inflammation	1
**Treatment alteration**	
Further anti-cancer treatment	2
Adding corticosteroids	4
Adding anti-fibrotic treatment	1

**Table 3:** Incidence of adverse events.

Events within 24 h Following Bronchoscopy Procedure	Numbers (*N* = 10)
No adverse events	7
Fever above 38 °C	2
Significant hypoxemia (oxygen saturations < 90% on FIO_2_ 1.0)	1
Tachycardia > 150 beats/min	0
Ventricular arrhythmia	0
Pneumothorax	1
Hemorrhage requiring blood transfusion	0
Death	0

The patient had pneumothorax and significant hypoxemia.

## Data Availability

Not applicable.

## Abbreviations

ARDS	Acute respiratory distress syndrome
DAD	Diffuse alveolar damage
FiO_2_	Fraction of inspired oxygen
PaO_2_	Partial pressure of oxygen
SpO_2_	Oxygen saturation
ICU	Intensive care unit
INR	International normalized ratio
PEEP	Positive end-expiratory pressure
SLB	Surgical lung biopsy
TBLB	Transbronchial lung biopsy
TBLC	Transbronchial lung cryobiopsy
